# Anisotropic mesoporous silica/microgel core–shell responsive particles†

**DOI:** 10.1039/d0ra02278k

**Published:** 2020-07-03

**Authors:** Julien Schmitt, Caroline Hartwig, Jérôme J. Crassous, Adriana M. Mihut, Peter Schurtenberger, Viveka Alfredsson

**Affiliations:** Division of Physical Chemistry, Department of Chemistry, Lund University 221 00 Lund Sweden; LSFC Laboratoire de Synthèse et Fonctionnalisation des Céramiques, UMR3080 CNRS/Saint-Gobain CREE, Saint-Gobain Research Provence 550 Avenue Alphonse Jauffret Cavaillon France Julien.Schmitt@saint-gobain.com; Institute of Physical Chemistry, RWTH Aachen University 52074 Aachen Germany crassous@pc.rwth-aachen.de; Lund Institute of Advanced Neutron and X-ray Science (LINXS), Lund University Lund Sweden

## Abstract

Hybrid anisotropic microgels were synthesised using mesoporous silica as core particles. By finely controlling the synthesis conditions, the latter can be obtained with different shapes such as platelets, primary particles or rods. Using the core particles as seeds for precipitation polymerisation, a crosslinked poly(*N*-isopropylacrylamide) (PNIPAM) microgel shell could be grown at the surface, conferring additional thermo-responsive properties. The different particles were characterised using scattering and imaging techniques. Small angle X-ray scattering (SAXS) was employed to identify the shape and porous organisation of the core particles and dynamic light scattering (DLS) to determine the swelling behaviour of the hybrid microgels. In addition, cryogenic transmission electron microscopy (cryo-TEM) imaging of the hybrids confirms the different morphologies as well as the presence of the microgel network and the core–shell conformation. Finally, the response of the particles to an alternating electric field is demonstrated for hybrid rod-shaped microgels *in situ* using confocal laser scanning microscopy (CLSM).

## Abstract 2

Hybrid anisotropic microgels with different morphologies were prepared using mesoporous silica particles as core and PNIPAM as shell. The shell thickness *d* and aspect ratio *ρ* were characterised notably *via* cryo-TEM (left) and DLS (right).

## Graphical abstract

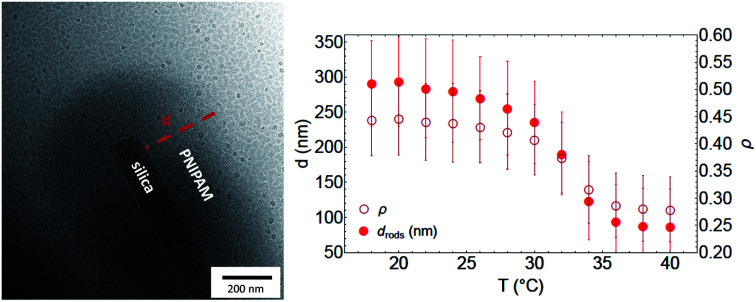

## 1 Introduction

Stimuli-responsive polymers, sensitive to pH or temperature,^1^ have attracted great attention from the scientific community, either for their possible applications such as drug carriers,^2–4^ or for their use as a model system for the study of self-assembly mechanisms^5,6^ or the phase behaviour of hard and soft colloids.^6–9^ Among them, poly(*N*-isopropylacrylamide) (PNIPAM) microgels are thermo-responsive polymers that undergo a hydrophilic to hydrophobic transition at a so-called “volume phase transition (VPT) temperature” *T*_VPT_ = 32 °C. Cross-linked PNIPAM microgels hence go from a swollen state in solution below *T*_VPT_ to a collapsed state above this temperature.^4,7,10,11^

By polymerising PNIPAM around an organic or inorganic core, core–shell structures can be obtained. As a few examples, PNIPAM microgel shells have been polymerised around another microgel,^12^ a polystyrene latex core,^13,14^ gold^15,16^ and silica^15^ particles. Core–shell microgels are versatile systems that combine the properties of the different constituents offering for instance appealing catalytic^14,17^ or optical properties^18^ related to the conformational control of the surrounding microgel shell with the temperature. While most of the past studies have been focusing on spherical composite microgels, there is an emerging interest for anisotropic microgels. The latter can be employed to explore complex self-assembly and dynamics^19–25^ considering that their size, anisotropy, softness and interaction potential can be finely varied with the synthesis conditions and the temperature. Various synthetic strategies have been involved to create such systems. The most prominent consists in employing functionalised anisotropic cores as seed for the microgel synthesis. Hereby, silica coated hematite^19^ and maghemite^20,26^ ellipsoidal particles have been implemented as magnetic core to create anisotropic microgels with tuneable orientation. Recently, it was even shown that the silica coated hematite core could be used as sacrificial template to the design of anisotropic hollow microgels.^27^ Other studies have shown that a similar approach could be applied to polymeric dumbbells^28^ or polystyrene ellipsoids.^25^ The second approach relies on the nanoengineering/post processing of composite microgels with a polystyrene core into various shapes such as ellipsoidal^21,22,29^ or bowl-shaped particles.^22–24^

Mesoporous silica materials, formed in solution by mixing a surfactant and a silica source,^30,31^ can be synthesised as colloidal porous particles and further used as inorganic templates in the synthesis of hybrid silica/PNIPAM particles. Most efforts have been made to graft the porous walls of the materials with PNIPAM, to allow access or blockage of the porosity for temperature-controlled drug delivery applications.^32–37^ Another approach is the formation of PNIPAM-coated silica particles. Spherical core–shell particles were synthesised, by grafting the PNIPAM *via* syntheses involving several steps, such as RAFT.^38,39^ Some of these hybrid particles exhibited drug-delivery potential,^40–43^ others were used in cellular imaging,^44^ anion exchange^45^ or as mini-reactors.^46^ In all cases, the main focus was to control the access to the inner porosity of the silica particles with the PNIPAM polymer shell.^47^

Among those mesoporous materials, SBA-15 is one of the most studied.^31,48^ This material has a 2D-hexagonal structure (plane group *p*6*mm*) with well-defined pores (diameter in the range of 5–10 nm). Moreover, recent studies show that it is possible to control, with precision, the morphology of the sub-micrometric mesoporous grains.^49,50^ Indeed, by controlling the synthesis conditions, such as temperature and stirring, it is possible to form SBA-15 particles with different shapes such as platelets,^51^ “rice grains”, hexagonal columns, short/long rods and even “donuts”.^52^ The versatility of the available morphologies makes this system extremely promising to create new and anisotropic core–shell silica/PNIPAM particles.

The aim of this paper is to present the synthesis of such particles, with thermo-responsive properties. The synthesis relies on simple seed radical polymerisation of NIPAM in presence of the silica core, without any surface modification of the silica particles. Indeed the formation of the hybrid particles is made possible by the inner porosity of the silica core which allows the crosslinked PNIPAM shell to grow around the silica particle during polymerisation. These new hybrid microgels have a high anisotropy, related to the shape of the porous silica core, with an aspect ratio depending on the temperature. They are, thanks to the PNIPAM shell, easily dispersible in suspension. Moreover, the presence of the silica core makes those particles highly polarisable to electric field, which can be used as an external trigger to control their orientation in suspension.

## 2 Materials and methods

### Anisotropic mesoporous silica synthesis

The syntheses of the mesoporous silica particles, used as the core in the hybrid particles, were carried out following already established protocols.^48,53^ The morphology of the material (SBA-15) is adjusted by control over the temperature and the stirring speed of the synthesis solution. Pluronics P123 ((EO)_20_-(PO)_70_-(EO)_20_) and P104 ((EO)_27_-(PO)_61_-(EO)_27_) were purchased from BASF and used as received. The silica precursors, either TEOS (tetraethyl-orthosilicate) or TMOS (tetramethyl-orthosilicate), were purchased from Sigma-Aldrich and used as received. SBA-15 particles were synthesised by adding the silica source (SI) to a pluronic (PL) solution at 2.5 wt% under acidic conditions (HCl 1.6 M). The synthesis solution had the following molar composition: HCl : H_2_O : PL : SI = 6 : 200 : 0.017 : 1. SBA-15 rods synthesis was performed at 50 °C, using P123 as the surfactant and TEOS as the silica source, with the stirring stopped after 5 min.^50^ The solution was then kept for 24 h at the same temperature. SBA-15 platelets were synthesised using P104 and TMOS at 55 °C under constant stirring for 24 hours, whereas the so-called primary particles were obtained under the same synthesis conditions but with the stirring stopped after one minute. After 24 hours of synthesis, solutions were placed at 80 °C for a 24 hours hydrothermal treatment. Afterwards the material was washed with ethanol, filtered and subsequently calcined at 500 °C for 6 hours.

### Hybrid microgel synthesis

The PNIPAM shell synthesis was done in presence of the silica core particles; using *N*-isopropylacrylamide (NIPAM) as monomer, *N*,*N*′-methylenebisacrylamide (BIS) as cross-linker, potassium persulfate (KPS) as initiator and methacryloxyethylthiocarbamoyl Rhodamine B (MRB) as dye for confocal microscopy imaging, all purchased from Sigma-Aldrich and used as received. In a synthesis, typically 100 mg or 500 mg of silica particles were dispersed in 50 mL of MilliQ water, using a sonication bath overnight (RM75U from Bandelin) to ensure proper dispersion. NIPAM was then added with the mass ratio of *x* = *m*_silica_/*m*_NIPAM_ = 0.51 for hybrid silica/PNIPAM platelets, *x* = *m*_silica_/*m*_NIPAM_ = 0.38 for primary particles and *x* = *m*_silica_/*m*_NIPAM_ = 0.25 for the rods. The value of *x* was chosen according to geometrical reasoning by calculating the amount of NIPAM to have a PNIPAM shell of roughly 200 nm in the swollen state. The cross-linker BIS was also added, with a molar ratio BIS : NIPAM = 5 : 100.^7^ Silica, NIPAM and BIS were mixed together in a reactor under nitrogen flow and stirred for 2 h at 60 °C to dissolve the monomer. The temperature was then increased to 80 °C for 30 min whereupon MRB (mass ratio MRB : NIPAM = 0.0016 : 1) was added using a 1 mg mL^−1^ solution in MilliQ water. Then, after *ca.* 5 min, KPS, the initiator for the polymerisation freshly dispersed in MilliQ water at 1 mg mL^−1^, was added dropwise using a syringe. The dropwise addition is used to promote shell growth and avoid secondary nucleation. A final mass ratio of KPS : silica = 0.025 : 1 was used, in agreement with previous syntheses of core–shell materials.^20^ The reaction was let to proceed for 4 hours at 80 °C under nitrogen flow. Finally the sample was filtered with glass wool and then centrifuged 3 times at 5000 rpm for 15 min.

### Characterisation

Mesoporous silica particles and hybrid silica/PNIPAM particles were studied using scanning electron microscopy (SEM) to characterise their morphology and with small angle X-ray scattering (SAXS) to check the morphology of the core and its inner pores structure. The silica/PNIPAM particles were also studied using SEM and cryogenic transmission electron microscopy (cryo-TEM) to evidence the presence of a PNIPAM shell, and dynamic light scattering (DLS) to estimate the shell dimensions from the particles translational diffusion coefficient measured at different temperatures. Finally, suspensions were studied using confocal laser scanning microscopy (CLSM) to observe their eventual response in dispersion to the application of an external alternating electric field.

### Scanning electron microscopy (SEM)

For the SEM measurements, dried samples were deposited on a stub, and then coated with approximately 15 nm gold/palladium to ensure electron conductivity of the sample. Measurements were carried out using a JEOL JSM 6700 at a voltage of 10 kV and a current of 15 mA.

### Cryogenic transmission electron microscopy (cryo-TEM)

To evidence the presence and conformation of the microgel shell, cryo-TEM measurements were carried out on a JEM-2200FS (JEOL, Japan), operated at 200 kV. The samples were vitrified in a controlled environment vitrification system, operating at 20 °C with a relative humidity close to 100%. A small volume (*ca.* 5 μL) of hybrid platelets, primary particles or rods at 0.2 wt% in MilliQ water, stored at room temperature, were deposited on lacey carbon-coated copper grids. After bloating the liquid excess, the grids were quenched into liquid ethane, and then stored in liquid nitrogen until being transfered to the electron microscope.

### Confocal laser scanning microscopy (CLSM)

Suspensions of hybrid silica/PNIPAM rods (2 wt%) were studied using an inverted confocal laser scanning microscope (CLSM) (Leica DMI6000) with an SP5 tandem scanner in the resonant mode using a 100× oil immersion objective. The CLSM was mounted in a thermostated enclosure, which enabled us to control the temperature with an accuracy of 0.2 °C. The microgel suspension (6 μL) was sandwiched between the coverslips and hermetically sealed by a 120 μm thick spacer with a 5.1 mm aperture (SecureSeal Imaging). Imaging under electric field was performed by coating one coverslip with approximately 15 nm gold/palladium deposit (same protocol than for stub coating in SEM), leaving a 1 mm band gap uncoated; and connecting the two half-coated parts to a wideband amplifier coupled with a frequency generator. An alternating electric field of 50 kV m^−1^ was applied at a frequency of 160 kHz. This setup allowed generating an electric field between the electrodes along the imaging plane.

### Small angle X-ray scattering (SAXS)

SAXS measurements were performed using a GANESHA SAXS system (SAXSLAB, Denmark), for “high” *q*-range (0.06–0.26 Å^−1^) in order to characterise the pores structure of the particles. In addition, measurements at the Paul Scherrer Institute (PSI) on the cSAXS beamline permitted to study a lower *q*-range (5 × 10^−4^ to 0.1 Å^−1^) in order to characterise the different morphologies. Suspensions of particles in water at 1 wt% were measured for 2 hours on the GANESHA and 3 seconds at PSI, and signals have been treated to remove the background contribution.

For mesoporous silica particles, the intensity *I*(*q*) can be described as the sum of two parameters:

1 *I*(*q*) = *I*_particle_(*q*) + *I*_Bragg_(*q*)

with *I*_particle_(*q*) the contribution of the overall particle at very small angles and *I*_Bragg_(*q*) the contribution from the porosity at larger angles, giving rise to Bragg peaks due to the 2D-hexagonal organisation of the pores.

**Fig. 1:**
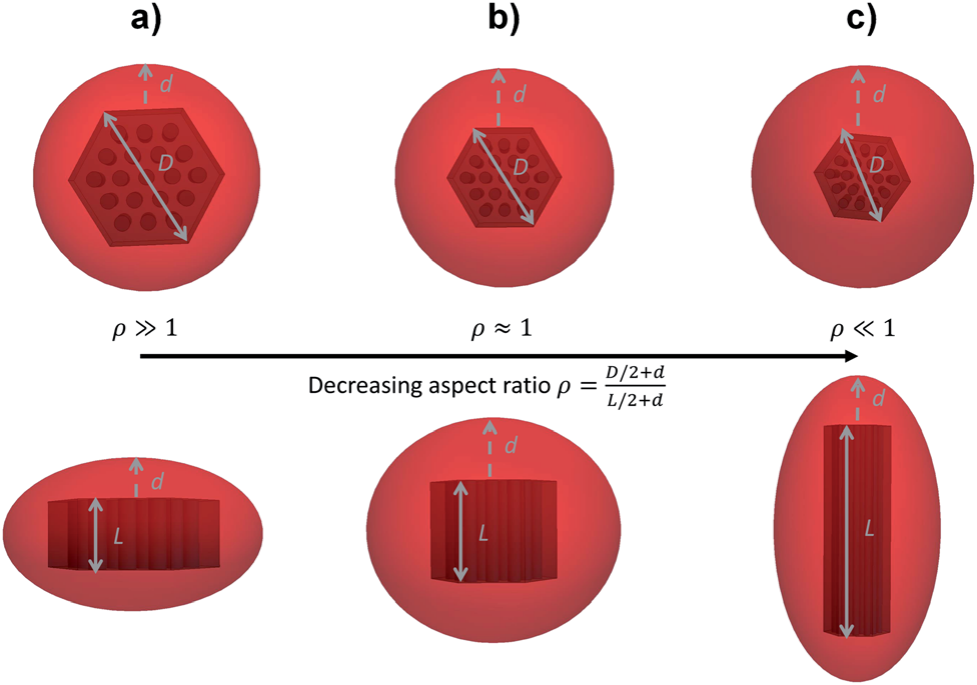
Sketch of hybrid silica/PNIPAM (a) platelets, (b) primary particles and (c) rods, both in top (top) and side (bottom) views. *D* is the diameter of the silica core, in the plane perpendicular to the porosity. *L* is the length of the core along the 2D-hexagonal porosity and *d* is the shell thickness of the hybrid silica/PNIPAM particles. For primary partiles (b), *D* ∼ *L* and are approximated in SAXS by a sphere of radius *R*.

The small angles of the data acquired in cSAXS were hence fitted using:

2 *I*_particles_(*q*) ∝ 〈|∑(*q*)|^2^〉_*σ*_

with 〈|∑(*q*)|^2^〉_*σ*_ the form factor of the particles (either using a platelet like model; a spherical model for primary particles or a rod-like model)^50^, which depends on the dimensions of the particles (the diameter *D* and length *L* for platelet and rod; and the radius *R* of the spheres for primary particles, see Fig. 1). 〈〉_*σ*_ characterises the polydispersity in size on the smallest dimension of the particle (*i*.*e.* the length *L* for platelets, the radius *R* for primary particles or the diameter *D* for rods), taken into account using the Schulz–Zimm distribution in size,^54^ with *σ* being the polydispersity index. Models of platelet, sphere and rod morphology are well-established in the literature; details of their use to fit mesoporous particles can be found in ref. 50 and are not provided here.

### Dynamic light scattering (DLS)

Suspensions of bare silica and hybrid silica/PNIPAM particles at 0.01 wt% were measured by DLS (Goniometer ALV/CGS-5022F, ALV Gmbh, Langen, Germany) upon changing temperatures (18 °C to 40 °C) and a scattering angle of 50° to measure the effect of temperature on the PNIPAM shell. Suspensions of hybrid silica/PNIPAM particles were also measured at 90° and 110° for a more accurate angular dependent determination of the translational diffusion coefficient *D*_t_. DLS data were treated using the second order cumulant analysis.^55,56^ For monodisperse particles, the first-order electric field correlation function is a single exponential:

3 *g*^1^(*τ*) = e^−*Γτ*^,

with a decay rate of *Γ* = *D*_t_*q*^2^. *D*_t_ is the translational diffusion coefficient (in m^2^ s^−1^) of the particle and *q* is the scattering vector, with *q* = 4π*n*/*λ* sin(*θ*/2), that depends on the scattering angle *θ* (°), the refractive index of water *n* = 1.332 and the laser wavelength *λ* = 632.8 nm. The variation of refractive index with temperature was neglected.

For polydisperse particles, the second order cumulant analysis allows writing *g*^1^ as a deviation from a monoexponential:4
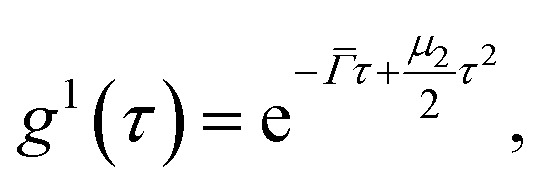
with *Γ̄* the average value of the decay rate and *μ*_2_ the second order moment. The polydispersity can be defined as 
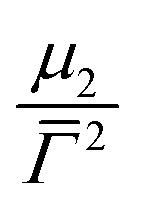
.

For spherical particles in a dilute dispersion, the translational diffusion coefficient *D*_t_ can be related to the particle hydrodynamic radius *R*_H_ through the Stokes–Einstein equation:5
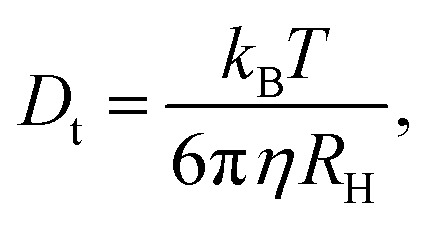
with *k*_B_ the Boltzmann constant, *T* the temperature in Kelvin and *η* the viscosity of the solvent (water). The variation of *η* with the temperature is taken into account. Fig. 1 shows the shapes of the hybrid silica/PNIPAM particles. All particles can be defined as core–shell, with a core dimension given by the diameter *D* and the length *L* for platelets (*D* ≫ *L*), primary particles (*D* ≥ *L*) and rods (*L* ≫ *D*); and a shell of thickness *d* that we assume constant. As the silica cores are not spherical, the DLS data of the hybrid particles were treated using models for oblate (for platelets and primary particles) and prolate (rods) ellipsoids. In that case, there is a correction factor *G*(*ρ*) to the diffusion coefficient:^57^6
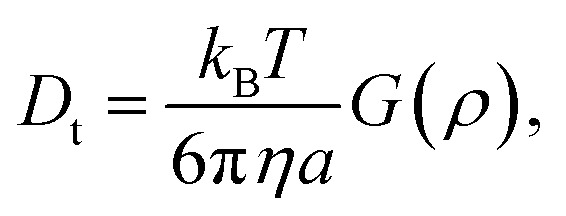
with *ρ* = *b*/*a* the aspect ratio of the particles, with *b* = *D*/2 + *d* the minor axis radius and *a* = *L*/2 + *d* the major axis radius. For prolate particles, *a* > *b* and *G*(*ρ*) is:7
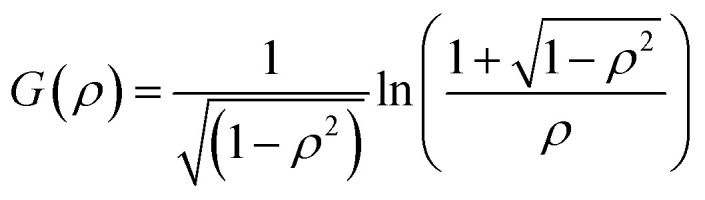


For oblate particles, *a* < *b* and this time *G*(*ρ*) reads:8
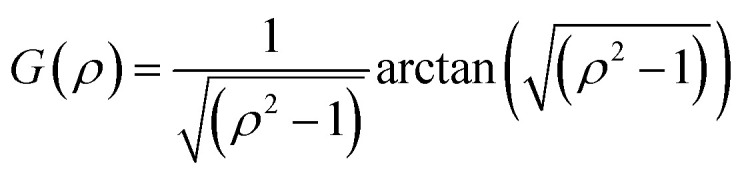


For anisotropic particles, two parameters are hence needed to describe them, the major axis radius and the minor axis radius (or the major axis radius and the aspect ratio of the particle). Alternatively, the particles can be defined by their core dimensions and shell thickness *d* (see Fig. 1). By knowing the core dimensions of the particles (from SEM measurements), it is then possible to estimate the thickness of the PNIPAM shell *d* and the overall aspect ratio *ρ*(*d*) of the particle as function of temperature. In both cases, the only adjustable parameter for our data evaluation is *d*.

## 3 Results and discussion

**Fig. 2:**
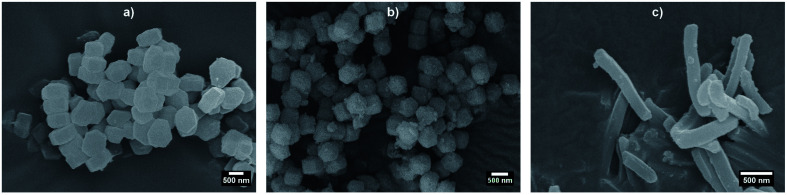
SEM micrographs of bare mesoporous silica (a) platelets, (b) primary particles, and (c) rods.

**Fig. 3:**
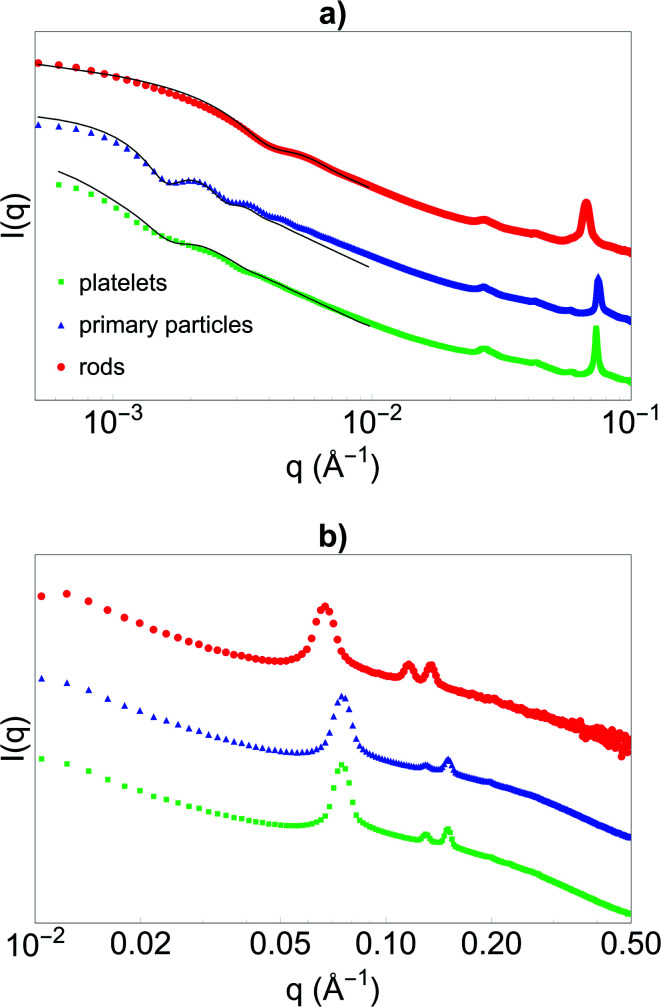
SAXS intensity *I*(*q*) *versus q* obtained with the (a) cSAXS beamline and (b) the GANESHA instrument of the silica (green) platelets, (blue) primary particles and (red) rods. Curves are shifted in intensity for clarity. In (a), fits of the shape of the particles using models for platelets, spheres and rods respectively are given as black lines.

Before synthesising the PNIPAM shell, the morphology and porous organisation of the mesoporous silica particles were checked using SEM (Fig. 2a–c for platelets, primary particles and rods respectively, and Fig. S1 in ESI†) and SAXS (Fig. 3). The dimensions of the particles were extracted from the SEM micrographs statistical analysis, whereas the fitting of the SAXS patterns (see Fig. 3a, and materials and methods for details concerning the fitting) gave the average length *L* of the platelets, the average radius *R* associated to the primary particles core and the average diameter *D* of the rods respectively, with their associated polydispersity. The highest dimension of the platelets and rods, reaching the micrometre scale, could not be measured in SAXS as it was not covered by the available *q*-range. The results show good agreement between the dimensions found in SEM and SAXS, the latter providing a better statistic. Moreover, Bragg peaks positions observed in SAXS (Fig. 3b) confirm the 2D-hexagonal organisation of the porosity and provide its lattice parameter. Parameters extracted from SEM and SAXS are gathered in Table 1.

**Table 1:** Structural data of the SBA-15 particles with different morphology types from SEM and SAXS

Particle	Platelets	Primary	Rods
*D* a (nm)	870 ± 90^d^	570 ± 50	210 ± 30
*L* a (nm)	390 ± 40	400 ± 40	1200 ± 400
*ρ* = *D*/*L*	2.2 ± 0.4	1.4 ± 0.3	0.18 ± 0.08
*X* b (nm)	*L* = 410 ± 5	2*R* = 540 ± 5	*D* = 180 ± 5
*σ* b (%)	19	12	20
*a*_0_ c (nm)	9.8 ± 0.1	9.8 ± 0.1	10.9 ± 0.1

a Dimensions measured by SEM (statistics over 100 particles with the error given by the standard deviation).

b Average size and polydispersity in size (*via* the Schulz–Zimm distribution) obtained by fitting the cSAXS data using the platelet, sphere (for primary particles) and rod models, respectively.

c Lattice parameter *a*_0_ obtained from the Bragg peaks positions on the SAXS data.

d Measured along the long diagonal of the hexagonally-shaped particles.

Dried hybrid silica/PNIPAM particles were also characterised using SEM (Fig. S2 and S3 in ESI†). SEM micrographs performed on dropcasted samples show that the silica cores are embedded in a PNIPAM matrix. The high concentration of dried particles (0.2 wt%) does not permit to observe isolated particles, as seen in Fig. S2.† The same samples, when dispersed *via* spin-coating techniques from a 0.01 wt% suspension (Fig. S3†), exhibit well-isolated particles.

**Fig. 4:**
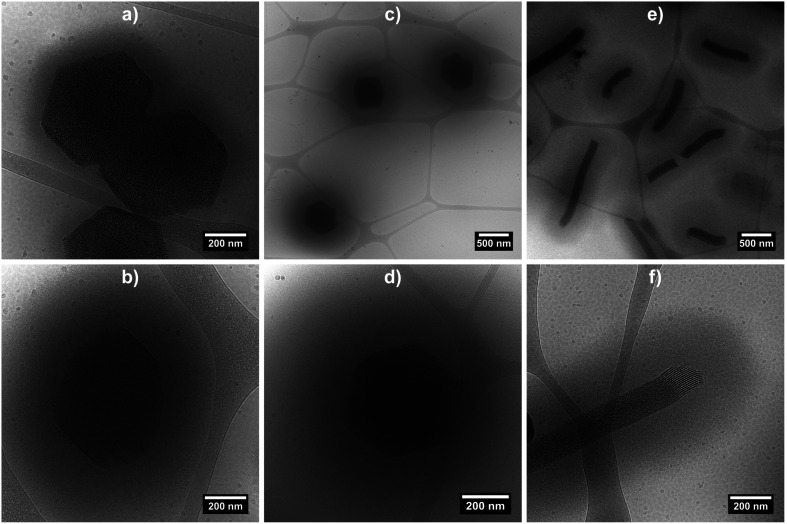
Cryo-TEM micrographs of hybrid silica/PNIPAM platelets (a and b), primary particles (c and d) and rods (e and f). Samples were prepared from a 0.2 wt% suspensions at 20 °C.

Cryo-TEM measurements were performed on the three hybrid morphologies (samples at 0.2 wt% at 20 °C) in order to image the swollen microgel shell (Fig. 4 and S4 in ESI†). The micrographs clearly evidence the porous organisation of the silica core, as well as the presence of a fuzzy PNIPAM shell, with shell thicknesses estimated at *ca.* 100 nm for hybrid platelets and 250 nm for both hybrid primary and rods particles, from 10 measurements.

**Fig. 5:**
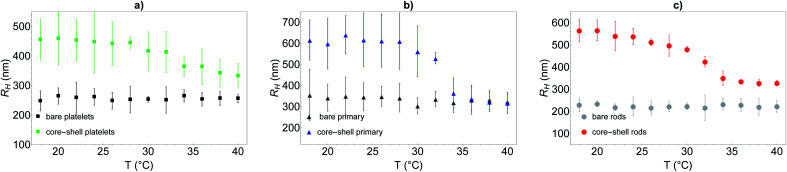
Hydrodynamic radius measured by DLS at 50° using the cumulant analysis over a wide temperature range (18 °C to 40 °C) for bare silica and hybrid silica/PNIPAM particles. Particles core morphologies are (a) platelet, (b) primary particle and (c) rod.

To further quantify the temperature influence on the shell conformation and on the overall morphology of the hybrid silica/PNIPAM particles, DLS measurements were carried out for temperatures ranging from 18 °C to 40 °C at a scattering angle of 50°, for both bare silica and hybrid silica/PNIPAM particles. Using the second order cumulant analysis of the data, the translational diffusion coefficient *D*_t_ was extracted (eqn (4)). Considering that platelets and rods are anisotropic, the swelling behaviour was determined by plotting the apparent hydrodynamic radius 
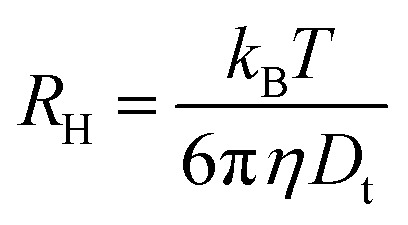
 (eqn (5)) of the equivalent sphere against the temperature. The presence of PNIPAM is further evidenced by comparing the bare silica particles with their hybrid silica/PNIPAM counterparts (Fig. 5). For the platelet morphology (Fig. 5a), the apparent hydrodynamic radius *R*_H_ is found constant for the bare silica platelets over the whole temperature range, with a value around 250 nm. Conversely, *R*_H_ becomes temperature dependent for its hybrid counterpart: it decreases above 32 °C, with a swollen *R*_H_ measured around 450 nm at low temperatures, and at 325 nm in the collapsed state at high temperatures. One can note that the measured polydispersity *σ* is higher for the hybrid particles than for the bare particles (*σ* = 18% below 30 °C on average for the hybrid platelets *versus* 14% for the bare platelets). The transition around 32 °C is consistent with *T*_VPT_ of PNIPAM microgels.^7^ A similar swelling behaviour is observed for all the investigated hybrid morphologies. At first sight, composite primary particles seems to exhibit the highest degree of swelling followed by rods and then platelets. This observation is consistent with the idea that the PNIPAM : silica ratio was adjusted to get the same shell thickness: hybrid microgels with a smaller core are hence expected to exhibit a larger relative variation of their dimensions across the VPT.

Let us note that the results presented in Fig. 5 assume a spherical morphology for the particles, and can hence only be used qualitatively. While the results demonstrate the temperature-dependence of the core–shell particles, a model closer to reality needs to be used if one hopes to obtain the shell thickness variation with temperature, especially for the platelet and rod-like particles.

**Fig. 6:**
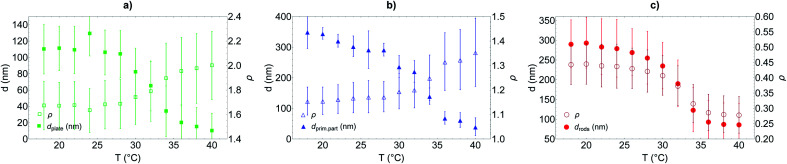
Shell thickness and aspect ratio associated to hybrid silica/PNIPAM (a) platelets, (b) primary particles and (c) rods *versus* temperature extracted from DLS measurements made at 50, 90 and 110° using an (a and b) oblate and (c) prolate model respectively; using SEM dimensions for the silica core. The error takes into account the error in measuring the diffusion coefficient in DLS and the size dimensions in SEM.

Hybrid particles were measured in DLS at three different angles (50°, 90° and 110°) with temperature, which allowed extracting with more accuracy the translational diffusion coefficient *D*_t_ by plotting *Γ̄* as a function of *q*^2^ (see Fig. S5 in ESI†). The hybrid particles can be considered in DLS as comprised of a solid silica core (either oblate for platelets and primary particles or prolate for rods; with dimensions obtained in SEM) plus a fuzzy shell thickness. Considering the average core dimensions from the SEM analysis and an oblate (for platelet and primary particles, eqn (6) and (8)) or prolate (rods, eqn (6) and (7)) model, it is possible to extract the shell thickness *d* and the aspect ratio *ρ* of the hybrid particles with temperature (see Fig. 6). Let us note that for platelets, we used a diameter 800 nm instead of 870, as it corresponds to the diameter of the equivalent platelet with a circular cross-section instead of a hexagonal one. Moreover, while a cylinder or spherocylinder model could have provided a more accurate description following the particle geometry, we have arbitrarily assumed an ellipsoidal shape due to the simplicity of the models.

It is observed that for the three morphologies, particles have a well-defined PNIPAM shell dependent on the temperature, and a transition temperature of 32 °C. In the swollen state, the shell thickness is above 100 nm for platelets and 250 nm for primary particles and rods (in good agreement with the cryo-TEM estimations), whereas it shrinks at high temperature to reach *ca.* 10, 30 and 80 nm for platelets, primary particles and rods respectively.

Primary particles, due to their weak anisotropy, show only a weak variation of their aspect ratio throughout the entire temperature range (from *ca.* 1.1 to 1.4). For platelets and rods, the shell thickness influences more strongly the effective aspect ratio of the particles. The aspect ratio *ρ* increases with the temperature for the platelets from *ca.* 1.6 to 2 while it decreases for the rods from *ca.* 0.44 to 0.28, in both cases indicating that the particles become more anisotropic as the shell collapses above *T*_VPT_.

From the analysis of the DLS data, it is clear that there is a difference between the thinner shell observed for the hybrid silica/PNIPAM platelets and the thick shell obtained for the two other morphologies. A possible explanation could lie in the interaction between NIPAM and the silica core during synthesis. The absence of mandatory surface modification to form these core–shell particles suggests NIPAM adsorption onto silica. The larger diameter *D* of the platelets would induce stronger plane–plane interaction between platelets in presence of NIPAM, with more aggregates formed (later removed during the glass wool filtration) and less overall NIPAM for dispersed particles. This hypothesis is supported by the fact that tests of syntheses made with platelets with an even higher aspect ratio *ρ* (3.9 *versus* 2.2 for the platelets presented here) aggregated at 60 °C before NIPAM polymerisation.

Moreover, it can be noted that for rods and primary particles, the shell thickness is found to be *ca.* 300 nm in the swollen state, instead of the 200 nm predicted based on the initial geometric reasoning. This mismatch can be explained by the presence of pores in the silica particles. The calculation assumed the pores would be uniformly filled with PNIPAM, in a similar conformation than the shell. The pores might be only partially filled, resulting in more NIPAM used to form the shell.

It is also worthwhile to note that bare silica mesoporous particles dispersed in water have the tendency to sediment and aggregate in water with time. The addition of the PNIPAM shell greatly promotes the stabilisation of the particles in water and allows for an easy redispersion without additional sonification.

**Fig. 7:**
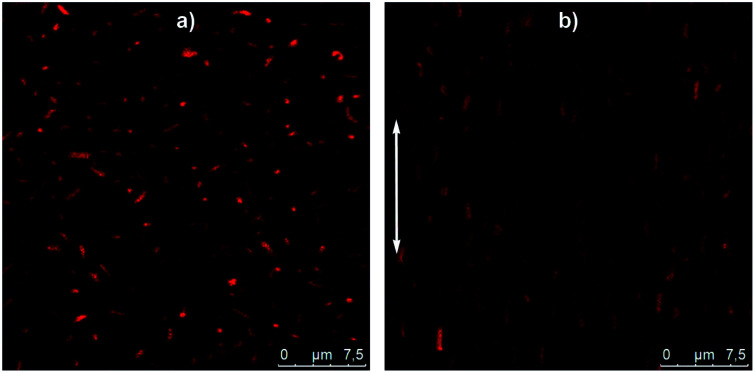
Confocal micrographs of a deionized suspension of hybrid silica/PNIPAM rods at 2 wt% (a) without electric field and (b) under an alternative electric field of *E* = 50 kV m^−1^ and *f* = 160 kHz. The white arrow gives the orientation of the electric field. Particles are dyed using MRB (methacryloxyethylthiocarbamoyl Rhodamine B).

Thanks to their micrometer size, the behaviour of these hybrids silica/PNIPAM particles in suspension can be monitored using CLSM. As an example, a 2 wt% hybrid silica/PNIPAM rod suspension has been studied (Fig. 7a and Video S1 in ESI†). Particles are clearly visible due to the presence of the labeled microgel shell and present a good dispersion in water and a random orientation distribution due to their free Brownian diffusion. Interestingly, the presence of silica does not only provide the inner anisotropy to the particles, but also makes the particles sensitive to electric fields. Indeed, the same suspension under an alternative electric field *E* = 50 kV m^−1^ at a frequency of *f* = 160 kHz shows a clear alignment of the rods along the applied field (Fig. 7b and Video S2 in ESI†). These results demonstrate the possibility to control the orientation of the particles in suspension using this external trigger. Coupled with their temperature-responsiveness and the adjustment of their anisotropy, this makes these particles promising building blocks for supracolloidal organisations.

## 4 Conclusion

Mesoporous SBA-15 particles have been used as core to form new core–shell silica/PNIPAM particles with tuneable anisotropy. SBA-15 can be synthesised as platelets, hexagonal columns or rods, and the hybrid particles retain a high anisotropy. The silica core morphology was determined from SEM and SAXS measurements, and the PNIPAM shell was evidenced by cryo-TEM and DLS studies. From the analysis of the DLS data, the shell thickness was extracted, showing a swelling/deswelling behaviour with temperature, and a *T*_VPT_ = 32 °C characteristic of PNIPAM. The shell reaches hundreds of nm below *T*_VPT_ and shrinks under 100 nm at high temperature. The presence of the PNIPAM shell allows the efficient dispersion of the particles in water, as evidenced by confocal microscopy. Confocal measurements showed that an electric field can be used as an external trigger to orient the particles in dispersions. Their excellent dispersability in water and responsivity to electric fields make these particles promising building blocks not only for fundamental studies, such as probing the phase diagram of platelet-like particles in suspension; but also for applied studies. A possible example would be their use to shape new organic/inorganic materials such as artificial nacres requiring additional control of the building block alignment. Furthermore, these particles, thanks to the porosity of the silica core, their easy functionalisation with PNIPAM and their biocompatibility, could be interesting carriers for drug-delivery applications.

## Conflicts of interest

There are no conflicts to declare.

## Supplementary Material

RA-010-D0RA02278K-s001

RA-010-D0RA02278K-s002

RA-010-D0RA02278K-s003
