# Supplementary material for: Anisotropic mesoporous silica/microgel core–shell responsive particles
Source: RSC Adv. 2020 Jul 3;10(42):25393–401. doi: 10.1039/d0ra02278k (PMC9055282; doi:10.1039/d0ra02278k)
Supplement: RA-010-D0RA02278K-s001 [file RA-010-D0RA02278K-s001.pdf]

Electronic Supplementary Material (ESI) for RSC Advances.
This journal is © The Royal Society of Chemistry 2020

Supplementary information

Anisotropic Mesoporous Silica/Microgel Core-Shell Responsive Particles

Julien Schmitt[a,b], Caroline Hartwig[a], Jérôme J. Crassous[a,c], Adriana M. Mihut[a], Peter Schurtenberger[a,d] and Viveka Alfredsson[a]

[a] Division of Physical Chemistry, Department of Chemistry, Lund University, 221 00 Lund, Sweden.
[b]LSFC Laboratoire de Synthèse et Fonctionnalisation des Céramiques UMR 3080 CNRS / Saint-Gobain CREE, Saint-Gobain Research Provence, 550 avenue Alphonse Jauffret, Cavaillon, France
[c] Institute of Physical Chemistry, RWTH Aachen University, 52074 Aachen, Germany,
[d] Lund Institute of advanced Neutron and X-ray Science (LINXS), Lund University, Lund, Sweden.

<u>Figure S1:</u> SEM micrographs of silica (a) platelets, (b) primary particles and (c) rods from powder deposition on the stub

(a)
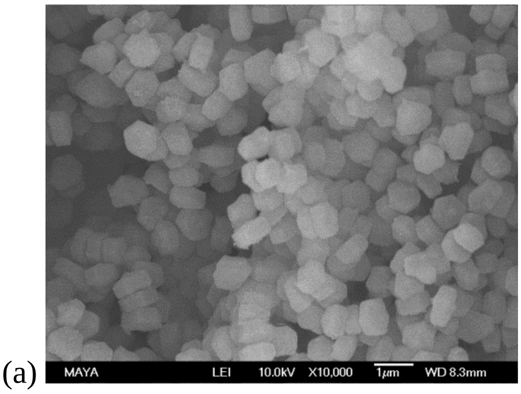

(b)
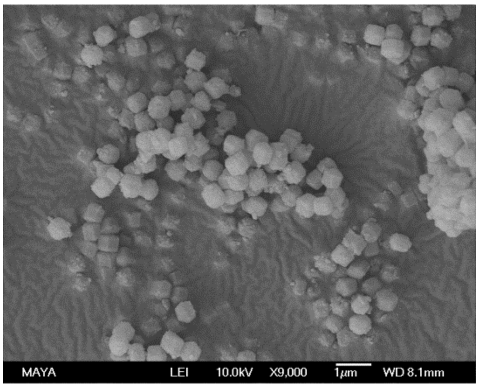

(c)
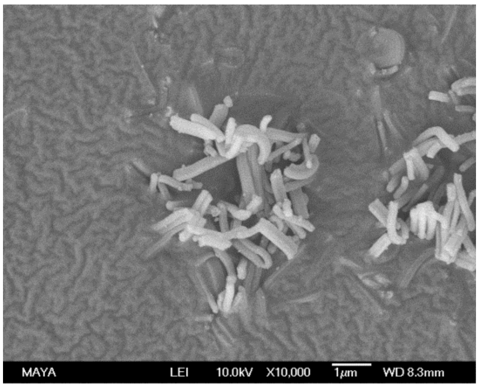

Figure S2: SEM micrographs of hybrid silica/PNIPAM (a) platelets, (b) primary particles and (c) rods from droplet deposition of a 0.2wt% suspension on a glass coverslip then deposited on the stub. The black arrows highlight the PNIPAM bridges between particles

(a) 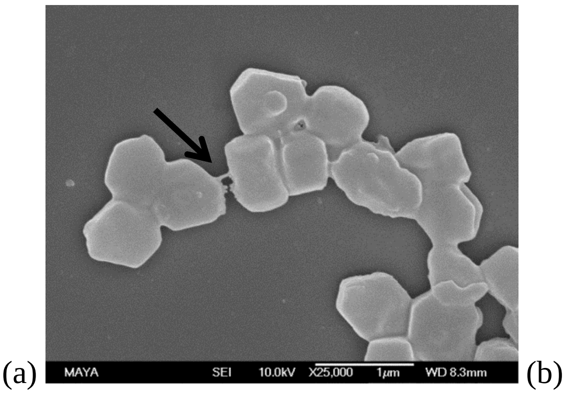

(b) 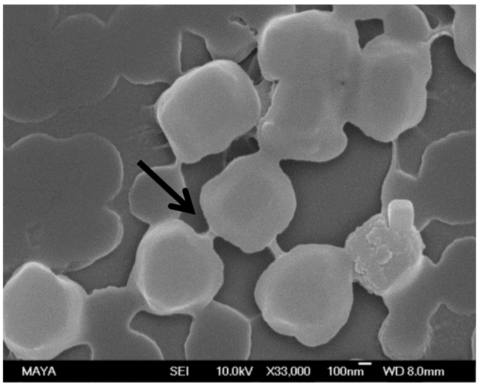

(c) 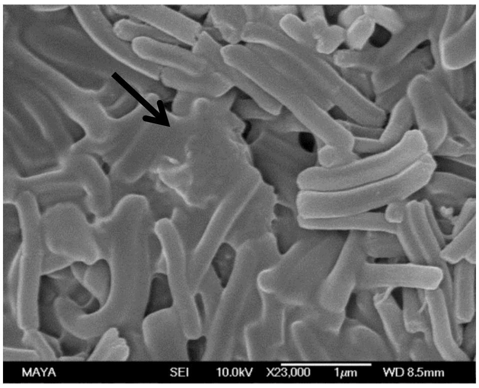

Figure S3: SEM micrographs of hybrid silica/PNIPAM (a) platelets, (b) primary particles and (c) rods obtained after spin-coating of suspensions of particles at 0.01 wt% on a glass surface.

(a) 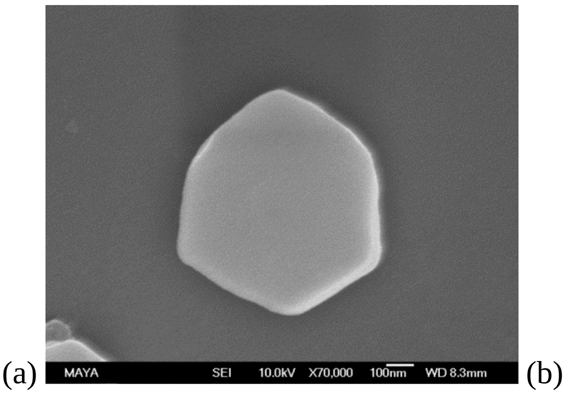

(b) 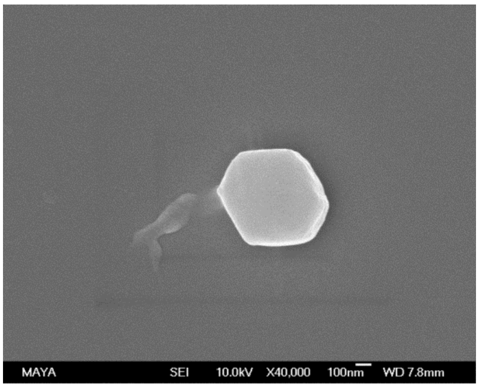

(c) 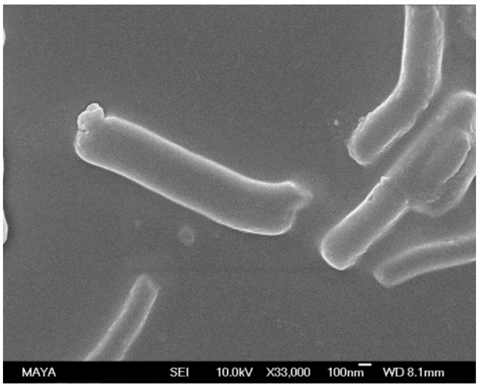

Figure S4: Cryo-TEM micrographs of hybrid silica/PNIPAM (a and b) platelets, (c and d) primary particles and (e and f) rods from dispersions at 0.2 wt% at 20°C.

(a) 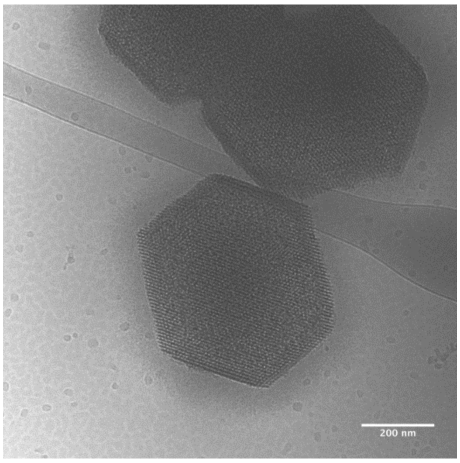

(b) 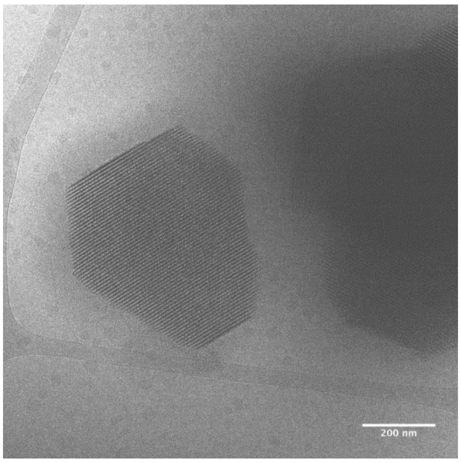

(c) 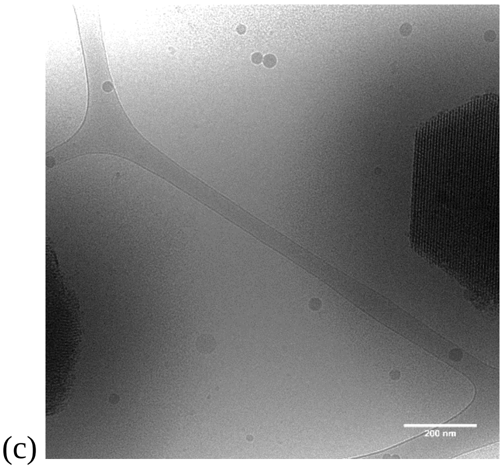

(d) 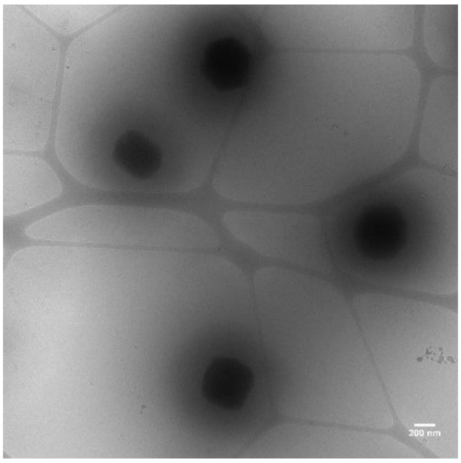

(e) 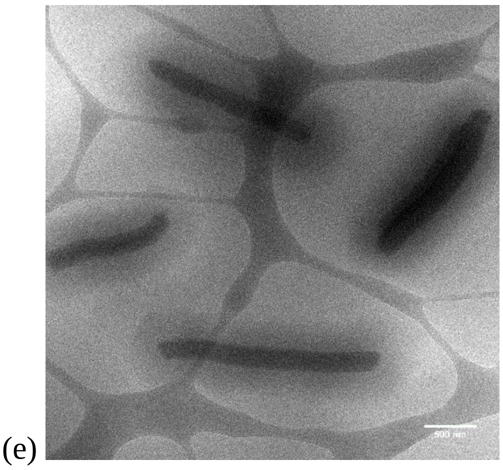

(f) 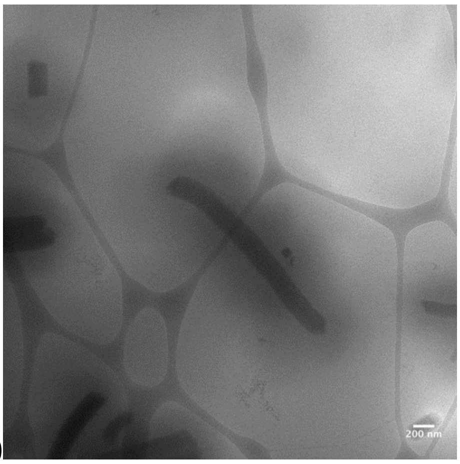

Figure S5: Plot of Γ versus q^2, with Γ extracted using the second order cumulant analysis of the correlation function $g^1(\tau)$, obtained from DLS measurements at 50° (q=11.18 μm^{-1}), 90° (q=18.70 μm^{-1}) and 110° (q=21.67 μm^{-1}) for hybrid silica/PNIPAM rods at 18°C. The fit in blue gives the translational diffusion coefficient D_t ($\mu m^2.s^{-1}$) ($\Gamma = D_t q^2$)

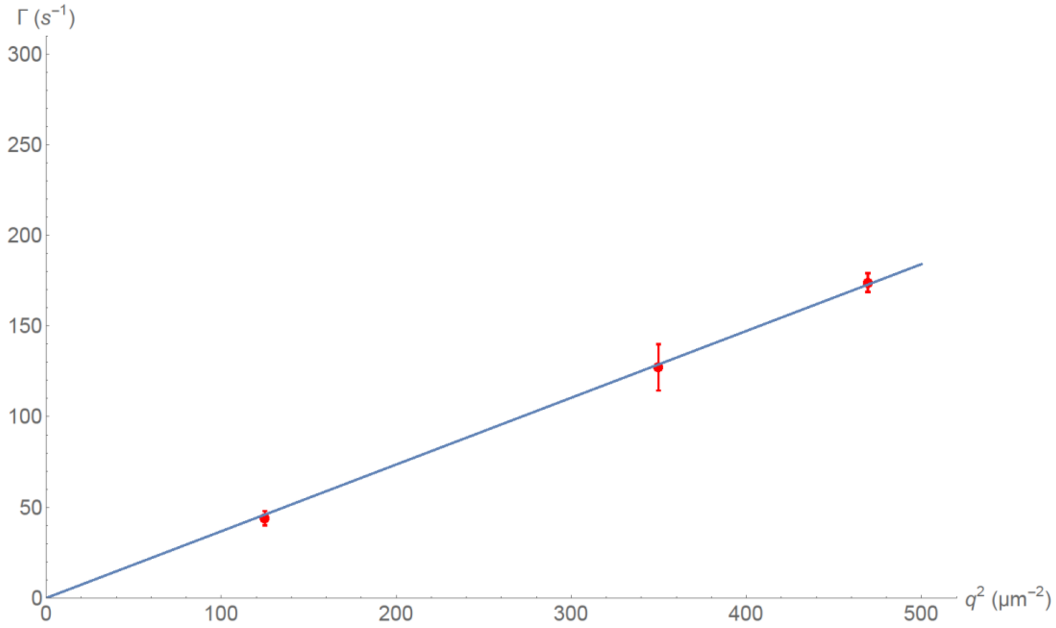

Video S1: CLSM video of a suspension made of hybrid core-shell silica/PNIPAM rods at 2wt% and 25°C without electric field

Video S2: CLSM video of a suspension made of hybrid core-shell silica/PNIPAM rods at 2wt% and 25°C under an alternate electric field, with a field strength E=50 kV/m and

frequency f=160 kHz. The rods are aligned along the direction of the field, along the imaging plane.
